# Four-dimensional flow assessment shows coronary artery flow reversal in severe aortic regurgitation

**DOI:** 10.1093/ehjcr/ytae059

**Published:** 2024-01-31

**Authors:** Mahesh Chandra Garg, Pankaj Garg

**Affiliations:** Department of Cardiology, Indraprastha Apollo Hospitals, New Delhi, India; Department of Cardiology, Norfolk and Norwich University Hospitals NHS Foundation Trust, Colney Lane, Norwich, NR4 7UY Norfolk, UK; Department of Cardiovascular and Metabolic Health, University of East Anglia, Research Park, Rosalind Franklin Rd, Norwich, NR4 7UQ Norfolk, UK

## Case description

**Figure 1:**
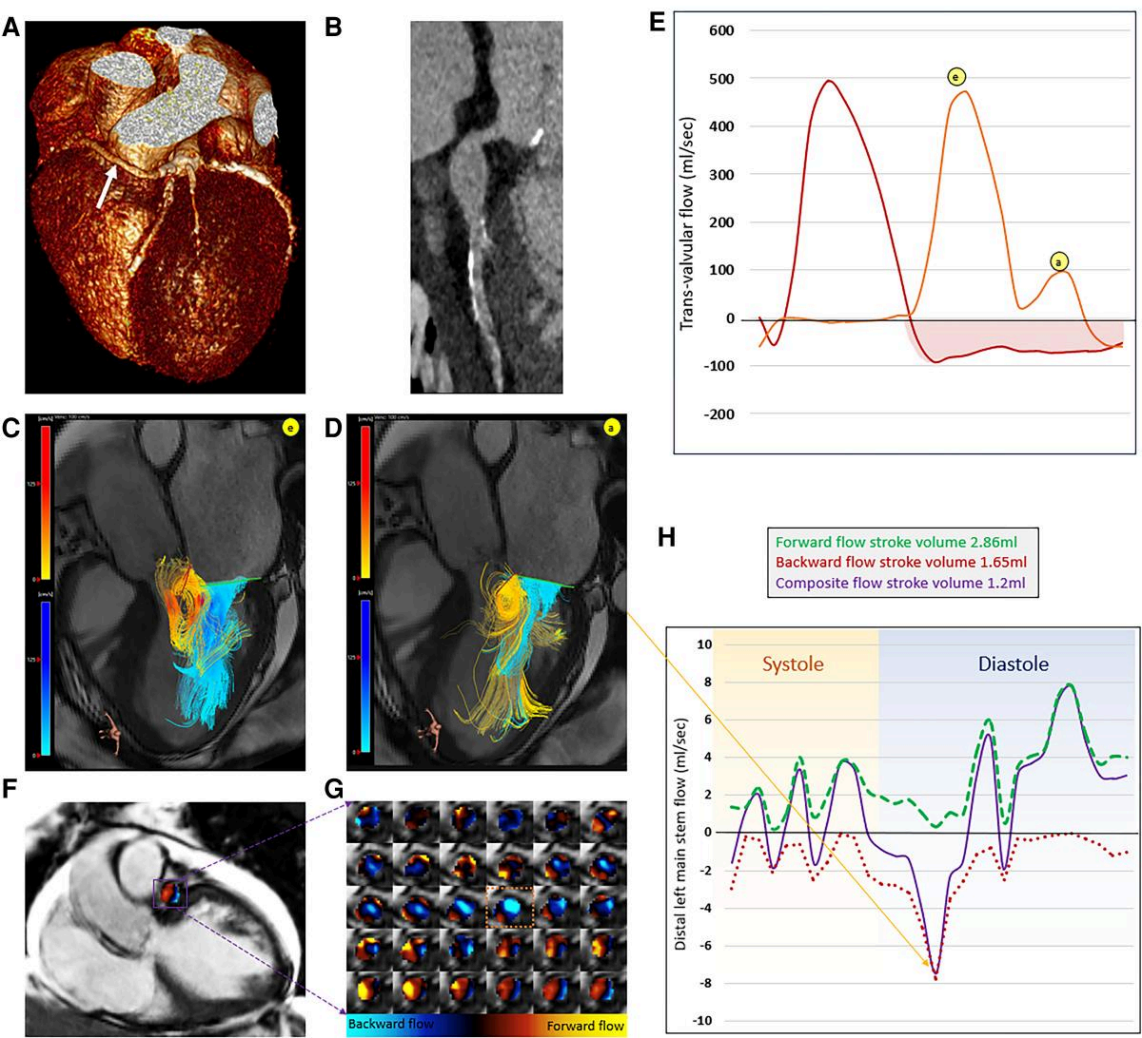
Four-dimensional flow imaging (4D flow) cardiovascular magnetic resonance imaging of aortic regurgitation and coronary artery. (*A*) Three-dimensional reconstruction of the heart and coronary arteries using cardiac computed tomography demonstrating that the right coronary artery originates from the left main stem (LMS) (white arrow). (*B*) Multi-planar reconstruction of the LMS and proximal left anterior descending artery demonstrating LMS dilatation. (*C*) Three-dimensional blood flow streamlines demonstrating competing aortic regurgitation (red-yellow scale) vs. mitral inflow blood flow (blue scale) during early filling. (*D*) Three-dimensional blood flow streamlines demonstrating competing aortic regurgitation (yellow) vs. mitral inflow blood flow (blue) during late filling. The aortic regurgitation interferes with the opening of the anterior mitral valve leaflet and restricts the filling. (*E*) Transvalvular flow curves (mitral, orange flow curve; aortic, red flow curve) computed using valve tracking procedures demonstrate significant holo-diastolic aortic regurgitation. (*F*) A coronal view cine where dilated left main step was identified and corresponding 4D flow data were then superimposed. (*G*) The LMS flow for the complete cardiac cycle (beginning with systole similar to flow curves in panel *E*) demonstrates backward flow during diastole as blue. (*H*) Coronary flow quantification flow curves demonstrate bi-phasic flow reversal during diastole—which is abnormal and mainly due to negative pressure in the aortic sinus, resulting from aortic regurgitation.

A 65-year-old man with a history of chest discomfort on exertion, breathlessness, and moderate aortic regurgitation (AR) was referred for cardiovascular magnetic resonance (CMR). A previous cardiac computed tomography showed that the left main stem (LMS) was aneurysmal (21 × 21 × 28 mm), and he had an anomalous coronary artery (right coronary origin from the LMS: benign course) (*Figure 1A*). Cardiovascular magnetic resonance examination was performed on a 1.5T Siemens Sola scanner (see Supplementary data). The CMR protocol included standard cines, T1 mapping, late gadolinium enhancement, and four-dimensional flow imaging (4D flow). For 4D flow, we used high spatial resolution (1.6 × 1.6 × 1.6 mm). The left ventricle (LV) was significantly dilated in end-diastole (134 mL/m^2^) and end-systole (78 mL/m^2^) with an LV ejection fraction of 44% (*Figure 1B*). The extracellular volume (ECV) was elevated at 34%.

On 4D flow streamline visualization, we observed that the AR was eccentric and thrashing towards the anterior mitral valve leaflet (*Figure 1C–E*), interfering with late LV filling. Aortic regurgitation quantified by valve tracking methods^1^ was severe, with a regurgitation fraction of 39%.^2^ A distal cross-sectional plane was used to quantify coronary flow (*Figure 1F–H*). We observed significant flow reversal during diastole. This flow reversal was bi-phasic. Normal coronary flow is ∼250 mL/min. In this gentleman’s case, it was significantly reduced to 60 mL/min, a total reduction of 74%.

In this case, cutting-edge 4D flow CMR imaging was used to unravel coronary flow physiology in a patient with severe AR, and this possibly explains the typical exertional symptoms of chest pain. This pathophysiological process has only been speculated previously in the literature.^3^ Future prospective studies are needed to establish the clinical prognostic role of myocardial ischaemia in the context of AR, where there is no significant coronary artery disease. Importantly, advancements in high-resolution 4D flow CMR imaging will likely broaden its clinical application—especially in coronary arteries.

## Supplementary Material

ytae059_Supplementary_Data

## Data Availability

The data underlying this article are available in the article and in its online Supplementary material.

## Supplementary material

**Consent:** The authors confirm that written consent for submission and publication of this case report, including images and associated text, has been obtained from the patient in line with Committee on Publication Ethics (COPE) guidance.

**Funding:** P.G. is funded by Wellcome Trust Clinical Research Career Development Fellowship (220703/Z/20/Z). For the purpose of open access, the author has applied a CC BY public copyright licence to any author accepted manuscript version arising from this submission. The funder had no role in study design, data collection and analysis, publication decision, or manuscript preparation.
