# Supplementary material for: Four-dimensional flow assessment shows coronary artery flow reversal in severe aortic regurgitation
Source: Eur Heart J Case Rep. 2024 Jan 31;8(4):ytae059. doi: 10.1093/ehjcr/ytae059 (PMC11017218; doi:10.1093/ehjcr/ytae059)
Supplement: ytae059_Supplementary_Data [file ytae059_supplementary_data.docx]

### Cardiac Magnetic Resonance protocol

CMR study was performed on a 1.5 Tesla Magnetom Sola Siemens system with a superconducting magnet (Siemens Healthineers AG, Erlangen, Germany). All patients were examined in the supine position, headfirst, using a respiratory sensor and electrocardiogram gating. Additionally, the scanner was equipped with an 18-channel biometric body coil and a built-in 32-channel spine coil.

The CMR protocol included baseline survey images and cines, gadolinium enhancement imaging, and four-dimensional (4D) flow acquisition methods previously described by our group (1–5). For standard cines, we acquired 30 phases throughout the cardiac cycle. Other cine acquisition parameters include TR: 2.71, TE: 1.13, field of view (FOV): 360 × 289.3mm2 with Phase FOV – 80.4%, number of signal averages (NSA): 1, matrix: 224 × 180 [phase], bandwidth: 167.4 kHz, [930Hz/Px], flip angle: 80, slice thickness: 8 mm and Grappa acceleration with a factor of 2.

**4D flow acquisition**

In the context of 4D flow acquisition, several specific imaging parameters were employed for the acquisition of cardiovascular data. The initial VENC (Velocity Encoded) setting was established within the range of 150 to 200 centimeters per second (cm/s) for both healthy controls (HCs) and individuals with Heart Failure with preserved Ejection Fraction (HFpEF). To capture the four-dimensional (4D) flow, 30 different time points or phases were acquired throughout the entire cardiac cycle, aligning the acquisition with the temporal consistency of cine (motion) data. The temporal resolution, representing the time interval between successive acquired phases, was set at 40 milliseconds (ms).

Additional acquisition parameters included the following:

- TR (Repetition Time): 4.98 milliseconds
- TE (Echo Time): 2.71 milliseconds
- Field of View (FOV): 200 × 256.3 square millimeters (mm²)
- Number of Signal Averages (NSA): 1, indicating that each image was acquired once
- Voxel Size: 3 × 3 × 3 cubic millimeters (mm³), representing the three-dimensional volume element used to represent the acquired data
- Bandwidth: 31.616 kilohertz (kHz) or 494 Hz per pixel (Px), indicating the range of frequencies captured in each pixel
- Flip Angle: 5 degrees, which represents the angle at which the magnetic spins are tipped to create the MR signal
- Grappa Acceleration: Employed in the phase-encoding direction with a factor of 2, implying that parallel imaging techniques were used to accelerate data acquisition
- Slice Direction: Set at 1, indicating the direction in which image slices were acquired within the three-dimensional space.

Furthermore, the electrocardiogram (ECG) data was retrospectively gated, meaning that it was synchronized with the cardiac cycle after data acquisition. This retrospective gating was performed during free breathing to avoid temporal blurring of diastolic phases, ensuring the accuracy of the acquired 4D flow data during the cardiac cycle.

**References**

1. Assadi H, Uthayachandran B, Li R, Wardley J, Nyi TH, Grafton-Clarke C, et al. Kat-ARC accelerated 4D flow CMR: clinical validation for transvalvular flow and peak velocity assessment. Eur Radiol Exp. 2022 Sep 22;6(1):46.

2. Barker N, Zafar H, Fidock B, Elhawaz A, Al-Mohammad A, Rothman A, et al. Age-associated changes in 4D flow CMR derived Tricuspid Valvular Flow and Right Ventricular Blood Flow Kinetic Energy. Sci Rep. 2020 Jun 18;10(1):9908.

3. Garg P, Crandon S, Swoboda PP, Fent GJ, Foley JRJ, Chew PG, et al. Left ventricular blood flow kinetic energy after myocardial infarction - insights from 4D flow cardiovascular magnetic resonance. J Cardiovasc Magn Reson Off J Soc Cardiovasc Magn Reson. 2018 Aug 30;20(1):61.

4. Garg P, van der Geest RJ, Swoboda PP, Crandon S, Fent GJ, Foley JRJ, et al. Left ventricular thrombus formation in myocardial infarction is associated with altered left ventricular blood flow energetics. Eur Heart J Cardiovasc Imaging. 2019 Jan 1;20(1):108–17.

5. Assadi H, Li R, Grafton-Clarke C, Uthayachandran B, Alabed S, Maiter A, et al. Automated 4D flow cardiac MRI pipeline to derive peak mitral inflow diastolic velocities using short-axis cine stack: two centre validation study against echocardiographic pulse-wave doppler. BMC Cardiovasc Disord. 2023 Jan 16;23(1):24.
